# Selective Laser Trabeculoplasty in Open-Angle Glaucoma and Ocular Hypertension: A Narrative Review of Current Evidence

**DOI:** 10.3390/jcm15197510

**Published:** 2026-09-27

**Authors:** Makedonka Atanasovska Velkovska

**Affiliations:** Department of Ophthalmology, University Medical Centre Ljubljana, 1000 Ljubljana, Slovenia; atmakedonka@yahoo.com; Tel.: +386-5147-4678

**Keywords:** selective laser trabeculoplasty (SLT), open-angle glaucoma, ocular hypertension (OHT), intraocular pressure (IOP), laser trabeculoplasty efficacy and durability, repeatability and re-treatment, safety and adverse events (IOP spikes inflammation), 180° vs. 360° SLT, emerging laser modalities (DSLT), micropulse laser trabeculoplasty (MLT), cost-effectiveness, real-world effectiveness

## Abstract

Selective laser trabeculoplasty (SLT) has emerged as a primary or adjunctive treatment choice for lowering intraocular pressure (IOP) in open-angle glaucoma (OAG) and ocular hypertension (OHT), addressing limitations of long-term topical therapy such as adherence challenges and cumulative ocular surface burden. This narrative review synthesizes current evidence on the mechanisms, efficacy and durability, safety, re-treatment, and predictors of response for SLT, with additional focus on treatment parameters (180° vs. 360°), emerging laser modalities (automated direct SLT/DSLT and micropulse laser trabeculoplasty/MLT), and economic and real-world effectiveness considerations. Mechanistically, SLT is based on selective photothermolysis of pigmented trabecular meshwork cells, aiming to minimize collateral structural damage and thereby support repeatability. Clinical outcomes from high-quality randomized evidence—most prominently the LiGHT program—demonstrate that SLT-first strategies achieve IOP control comparable to initial eye drops, with important patient-centered benefits including greater “drop-free” persistence, reduced medication burden, and lower need for incisional surgery over extended follow-up. However, durability attenuates over time in both trial and real-world settings, and long-term control may require escalation or re-treatment. The safety profile is characterized mainly by expected transient anterior segment inflammation and early IOP spikes, while rare vision-relevant complications (e.g., cystoid macular edema) have been reported, particularly in higher-risk clinical contexts such as prior intraocular surgery. Evidence supports meaningful repeatability, with a second SLT session showing the strongest support; data beyond later cycles remain more limited. Across studies, baseline IOP is the most consistent predictor of success, while factors such as fellow-eye response and trabecular angle pigmentation may influence both effectiveness and spike risk. Comparative evidence suggests that 360° SLT generally provides stronger early IOP reduction than 180° SLT, with differences becoming less consistent over longer follow-up. Emerging modalities may improve workflow efficiency (DSLT) and procedural tolerability (MLT), though larger datasets and longer-term durability evidence are still needed. Finally, cost-effectiveness analyses—especially when SLT is used as first-line therapy—support favorable economic outcomes driven by reduced or delayed ongoing medication use. Overall, SLT is well positioned as a cornerstone of modern glaucoma care, with optimal outcomes dependent on appropriate patient selection, parameter choice, risk-adapted follow-up, and safe re-treatment planning.

## 1. Introduction

Reducing intraocular pressure (IOP) is still the most important modifiable strategy to slow glaucoma progression and protect vision. Yet topical first-line therapy has practical limitations: it requires long-term daily use, frequent dosing, and continuous attention to ocular and systemic tolerability, while real-world adherence can be inconsistent. Over time, these factors can weaken treatment control.

Selective laser trabeculoplasty (SLT) therefore provides a valuable procedure-based alternative for lowering IOP without relying on patient compliance with daily eye drops. SLT was first introduced in 1995 as an advance in laser trabeculoplasty and gradually replaced argon laser trabeculoplasty (ALT). While ALT reduces IOP mainly through thermal injury and structural damage within the trabecular meshwork (TM), SLT is designed to target pigmented TM cells selectively. This selectivity is based on selective photothermolysis: brief laser pulses of approximately 3 ns concentrate heat in melanin-containing cells, limiting injury to adjacent non-pigmented TM tissue [1,2]. In line with this concept, SLT targets pigmented TM cells without producing structural TM damage, supporting both its favorable safety profile and the possibility of repeat treatment [1,2].

In recent years, SLT’s clinical role has strengthened substantially. In the LiGHT program, SLT was compared with IOP-lowering eye drops as first-line therapy in treatment-naïve open-angle glaucoma (OAG) and ocular hypertension (OHT). At three years, SLT was clinically effective and cost-effective, and many participants remained drop-free [3]. Six-year follow-up further confirmed sustained benefits, including better long-term disease control and a lower need for incisional glaucoma surgery and cataract procedures compared with initial drop therapy [4]. These results contributed to guideline updates and increasing recommendations for SLT as an initial treatment option alongside medication.

Despite these advantages, SLT is not curative. While SLT can achieve clinically meaningful IOP reductions, the magnitude of the IOP-lowering effect may diminish over time in some patients, and individual responses vary. Success rates can therefore change over time. For this reason, clinicians need a clear understanding of the mechanism, strength of the evidence, treatment parameters, and real-world performance—so that appropriate patients can be selected, durability can be anticipated, and escalation can be planned when needed. This narrative review synthesizes current evidence on SLT in OAG and OHT, with a focus on its role in modern treatment pathways and on emerging protocols that may improve predictability and routine clinical practice.

## 2. Materials and Methods

This is a narrative (non-systematic) literature review of selective laser trabeculoplasty (SLT) in open-angle glaucoma (OAG) and ocular hypertension (OHT). In addition, we also included evidence from secondary open-angle glaucomas—pseudoexfoliative glaucoma (PXG), normal-tension glaucoma (NTG), and pigmentary glaucoma (NTG)—to support and contextualize the subsequent discussion and to facilitate better understanding of some findings in POAG and OHT. This inclusion was limited to studies that reported relevant clinical outcomes (e.g., IOP reduction, safety, and durability/efficacy over time) and where the technical characteristics of SLT (e.g., laser parameters and treatment approach) were sufficiently comparable across studies. The review summarizes evidence related to mechanisms, efficacy and durability, safety, re-treatment, treatment parameters, predictors of response, real-world outcomes, emerging technologies, and cost-effectiveness, structured to match the domains reported in the Section 3.

We searched for studies published between January 2019 and July 2026 to capture contemporary evidence following the clinical and guideline influence of the LiGHT trial (2019). When the literature within this time window was insufficient for specific topics (e.g., foundational mechanistic concepts or key safety considerations), we included earlier seminal studies to support interpretation. To ensure broad coverage of clinical trials and real-world evidence, we searched the following bibliographic databases (as available to standard biomedical users): PubMed/MEDLINE, Embase, Cochrane Library, Scopus, Web of Science.

Search terms combined the following concepts (adapted to each database’s indexing language):

- Condition/population: open-angle glaucoma, ocular hypertension;
- Intervention: selective laser trabeculoplasty, SLT, laser trabeculoplasty;
- Comparisons/parameters: 180° vs. 360°, first-line vs. repeat SLT;
- Outcomes: intraocular pressure reduction, durability, treatment success, re-treatment, repeatability, medication use;
- Safety: intraocular pressure spike, inflammation, cystoid macular edema (CME);
- Predictors: baseline IOP, pigmentation/angle pigmentation, fellow-eye response;
- Emerging modalities: automated direct SLT (e.g., DSLT), micropulse laser trabeculoplasty (MLT);
- Economics (where available): cost-effectiveness, QALY, healthcare utilization.

Searches were run using combinations of these terms and their synonyms, with attention to retrieving both randomized and observational evidence relevant to SLT.

We included studies if they:

- Evaluated SLT in OAG and/or OHT (including repeat sessions when reported);
- Reported one or more outcomes relevant to the review (efficacy/durability, safety, predictors, or economic impact);
- Were clinical evidence (randomized trials, cohort studies, registry/real-world studies, and high-quality evidence summaries when necessary to contextualize findings).

Titles and abstracts were screened for relevance, followed by full-text review when needed. Evidence was synthesized narratively and thematically (not meta-analytically), organizing findings to align with the Results Subsections. Differences between studies (e.g., definitions of success, follow-up length, baseline characteristics, and re-treatment protocols) were discussed as sources of heterogeneity. Anthropic Claude was used to support language editing, rephrasing, and formatting to improve readability.

## 3. Results

In this narrative review, we included 53 references evaluating selective laser trabeculoplasty (SLT) in open-angle glaucoma (OAG) and ocular hypertension (OHT). The evidence base was drawn from publications dated January 2019 to July 2026 and studies published in or before 2018 (≤2018). Overall, the included set comprised 45 references from January 2019 to July 2026 and eight references from ≤2018, reflecting both established and more recent developments in efficacy, safety, re-treatment, treatment parameters, and real-world implementation (Table 1).

Across the included studies, SLT efficacy is primarily supported by (i) comparative evidence from randomized trials and (ii) effectiveness and safety data from real-world cohorts. The review also synthesizes evidence on durability of response, including time to failure and the clinical rationale for re-treatment, and summarizes safety findings with particular attention to expected short-term reactions (e.g., transient inflammation and IOP spikes) and less common but clinically relevant adverse events. Finally, the evidence includes evaluations of treatment parameters (e.g., treated angle extent), predictors of response, and more recent translational and implementation-oriented work, including emerging laser modalities and cost-effectiveness considerations.

### 3.1. Mechanism of Action and Technical Aspects of SLT

Selective laser trabeculoplasty (SLT) aims to lower intraocular pressure (IOP) in open-angle glaucoma by interacting selectively with the trabecular meshwork (TM), rather than causing broad structural ablation. Although the full mechanism is not completely established, converging evidence supports the idea that SLT improves aqueous outflow by altering TM cellular activity and triggering downstream biological changes in TM and Schlemm’s canal (SC) tissues [5,6]. Technically, SLT achieves this selectivity by using short Q-switched laser pulses at a wavelength that preferentially interacts with melanin-containing structures within the TM, reducing collateral thermal injury to nearby non-pigmented TM components [1,5].

#### 3.1.1. Selective Photothermal Targeting of Pigmented TM Cells 

Latina and Park demonstrated in vitro that selective targeting of pigmented TM cells is feasible under specific pulse duration conditions, using pulsed laser regimens intended to confine absorption to melanin-rich elements. In mixed cultures containing both pigmented and non-pigmented TM cells, the authors evaluated Q-switched Nd:YAG systems, frequency-doubled Nd:YAG, and other pulsed laser sources to identify a regime where absorption—and therefore damage—would remain confined to pigmented cells without collateral thermal or structural injury to adjacent non-pigmented TM elements [1].

A central finding was that pulse duration defines the boundary between selective and non-selective cellular injury. Selective targeting occurred with pulse durations between 10 ns and 1 µs, including 1 µs, with no collateral thermal or structural damage to neighboring non-pigmented TM structures under light microscopy and transmission electron microscopy [1]. In contrast, pulse durations longer than 1 µs caused non-selective killing of non-pigmented TM cells, consistent with increased heat diffusion and reduced melanin confinement [1].

The selective regime was also dose-dependent. The threshold radiant exposures were reported as low as 18 mJ/cm^2^, with threshold energy increasing when using longer wavelengths, longer pulse durations, and lower melanin content [1]. Together, these findings support the photobiological rationale of SLT: when the laser pulse is sufficiently short relative to thermal relaxation time and melanin absorption is adequate, the delivered energy is more likely to stay localized to pigmented TM elements [1,5].

#### 3.1.2. SLT Laser Parameters Used in Clinical Systems (And Why They Matter)

In clinical practice, SLT is typically delivered using a 532 nm frequency-doubled Q-switched Nd:YAG laser. Kennedy and colleagues connect key clinical parameters to selectivity: SLT uses a 400 µm spot size and a 3 ns pulse duration, with treatment energies in commonly available platforms typically in the range 0.3–2.0 mJ [5]. Selectivity is preserved because the pulse duration remains short relative to thermal relaxation time for melanin granules, thereby sparing surrounding non-pigmented cells [5]. This clinical parameterization matches the directionality from the in vitro pulse duration findings: short pulses support selective injury, while longer pulses promote non-selective effects [5].

#### 3.1.3. Cellular and Tissue-Level Consequences: From Local Stimulation to Outflow Remodeling

While the precise causal chain remains debated, several lines of evidence suggest that SLT-induced microdamage to pigmented TM cells is linked to downstream biological cascades that enhance outflow facility. Latina and Park support the initial trigger—localized melanin-related cellular injury under selective pulse conditions [5]. Downstream, SLT appears to induce remodeling and inflammatory/immune-related signaling within the TM and possibly SC tissues, offering a plausible pathway to sustained IOP lowering [1,6].

At the molecular level, Chen and Zeng describe mechanisms that include induction of immune and inflammatory responses in the TM (potentially related to oxidative damage), extracellular matrix remodeling, recruitment/activation of immune cells such as monocytes, and changes in SC permeability linked to junction disassembly [6]. These themes align with a multifactorial mechanistic framework in which SLT improves outflow through the TM, inflammation-driven remodeling of the extracellular matrix, and functional or proliferative TM cell changes [5].

Chen and Zeng also report experimental observations relevant to SLT biology: SLT may increase gene expression involved in matrix degradation while downregulating genes responsible for extracellular matrix formation, consistent with a remodeling phenotype [6]. They further describe associations between SLT and increased cytokine synthesis and secretion (including IL-1α, IL-1β, TNF-α, and IL-8), creating a mechanistic bridge between localized laser exposure and longer-term outflow changes [6].

#### 3.1.4. Why “Selective Thermolysis” Translates to Repeatability and Minimal Structural Collateral Damage

A consistent mechanistic theme is that SLT’s selectivity limits extensive structural disruption of the TM architecture compared with older argon-based approaches. Kennedy and colleagues emphasize that, compared with argon laser trabeculoplasty (ALT), SLT causes substantially less structural damage to the TM and Schlemm’s canal, with histopathologic and imaging studies demonstrating only minor disruption after SLT [5]. This reduced collateral injury is frequently used to justify clinical repeatability: the TM remains sufficiently intact to permit re-treatment if additional IOP lowering is required.

### 3.2. Efficacy of SLT: Short- and Long-Term Outcomes

#### 3.2.1. Short-Term Efficacy

Selective laser trabeculoplasty (SLT) demonstrates robust short-term IOP-lowering efficacy in both treatment-naïve OHT/OAG groups and in patients treated to reduce or replace topical therapy.

The pivotal multicenter LiGHT randomized controlled trial included 718 participants with newly diagnosed OHT or OAG. Participants were randomized to initial 360° SLT versus initial topical prostaglandin analog (PGA) therapy. At 36 months, the proportion of eyes achieving target IOP without additional treatment was 74.2% in the SLT arm and 78.2% in the medication arm (*p* = 0.22), indicating broadly comparable efficacy for IOP control [7]. Importantly, SLT enabled many participants to remain drop-free: a total of 74.2% of the eyes did not need eye drops to maintain target IOP at 36 months. Escalation to incisional surgery was markedly less frequent after SLT where zero eyes receiving SLT required incisional glaucoma surgery compared with 11 eyes in the medication arm [7].

Earlier comparative data also support SLT efficacy. McIlraith et al. reported mean IOP reductions of about 31% at 12 months after SLT, comparable to latanoprost monotherapy (mean reduction of 31.4%), with no statistically significant difference between groups [8]. In treatment-naïve cohorts with arc-length variations, Nagar et al. found mean IOP reductions of roughly 30% following 360° SLT at 12 months [9].

For patients already receiving topical medications, efficacy can be more variable but still clinically meaningful. A systematic review/meta-analysis by Rolim de Moura et al. reported a weighted mean IOP reduction after SLT of 5.09 mmHg (~22%) at 6 months in eyes on concomitant therapy. Success at 12 months varied according to the definitions used and the characteristics of included populations [10].

#### 3.2.2. Long-Term Efficacy (≥3 Years)

Long-term efficacy is best characterized through randomized follow-up data and supported by real-world effectiveness studies.

#### 3.2.3. LiGHT’s 6-Year Durability: Disease Control and Escalation Avoidance

Long-term outcomes from the LiGHT trial extension reported by Gazzard et al. at 72 months demonstrated sustained IOP-lowering benefit, with additional clinically important differences. A significantly lower proportion of eyes in the SLT arm required incisional glaucoma surgery compared with the medication group (0.5% vs. 3.8%, *p* = 0.004) [4]. Visual field progression and optic disk changes were broadly similar across arms, supporting the interpretation that SLT provides durable disease control rather than merely transient IOP reduction [4].

#### 3.2.4. Drop-Free Persistence and Medication-Sparing Effect

LiGHT also showed that SLT-first care can sustain a low treatment burden over years. At 36 months, 74.2% of the eyes in the SLT arm remained independent of drops, maintaining target IOP. At 72 months, the proportion of eyes remaining at or below target IOP without medical or surgical treatment remained substantial in the SLT group (69.8%), reinforcing SLT’s ability to reduce dependence on ongoing therapy [4].

#### 3.2.5. Surgery Avoidance Beyond Incisional Glaucoma Surgery (Cataract Surgery)

Within the longer-term LiGHT evidence set, SLT-first care was also associated with reduced overall escalation to surgical interventions, including cataract surgery. In the 6-year extraction of LiGHT, cataract surgery occurred more often in the medication group (95 eyes) than in the SLT group (57 eyes, *p* = 0.03), supporting a broader reduction in treatment escalation [4].

#### 3.2.6. Longer Follow-Up in Real-World Cohorts and Progression Signals

A retrospective study by Swain et al. with 3- and 5-year follow-up of 51 eyes treated with adjuvant SLT found that mean IOP decreased significantly in eyes that did not require additional surgical intervention, with reductions of 3.2 mmHg at 3 years and 3.5 mmHg at 5 years compared with baseline. However, disease control over the long term was not complete. At 5 years, the mean number of glaucoma medications increased significantly (2.7 ± 1.6) versus pre-SLT (2.0 ± 1.1, *p* = 0.04), suggesting a growing need for medical therapy over time. Consistent with this pattern, functional and structural progression was observed despite IOP reduction. On Humphrey’s visual field testing, mean deviation worsened at 5 years (−7.64 ± 6.57 dB) compared with baseline (−5.61 ± 3.90 dB), and pattern standard deviation increased significantly at both 3 and 5 years. On OCT, significant thinning of the inferior quadrant RNFL was observed at 5 years compared with baseline. Overall, these findings indicate that although SLT delivers sustained IOP lowering, it may not fully prevent long-term functional and structural progression when patients continue to require medication escalation [11].

A systematic review and meta-analysis by Chen et al. evaluated the long-term efficacy and safety of SLT in adult patients with primary open-angle glaucoma (POAG). The analysis included 60 peer-reviewed clinical studies (meta-analysis of 50 studies, 8934 eyes) with follow-up durations from 12 to 60 months. Across available evidence, SLT produced IOP reduction comparable to topical medications during the 6–12-month period (pooled mean difference of 0.04 mmHg, 95% CI: −0.27 to 0.34), indicating similar effectiveness early after treatment. Importantly, SLT reduced medication burden over the long term, with a pooled average decrease of approximately 0.87 glaucoma medications per patient (95% CI: −1.13 to −0.62) [12]. Functional outcomes supported these efficacy findings, with pooled analysis showing improvement or stabilization of visual field mean deviation by 0.79 dB (95% CI: 0.76 to 0.82) [12]. Safety signals indicated minimal acute IOP spikes and a generally favorable safety profile, with adverse events mainly limited to transient effects such as anterior chamber inflammation and short-term pressure changes [12].

#### 3.2.7. Real-World Long-Term Effectiveness and Re-Treatment Needs

Real-world effectiveness studies generally show attrition of SLT success over time, though outcomes remain clinically meaningful in a subset of eyes.

In routine UK EMR practice, Khawaja et al. analyzed 831 eyes after the first SLT and demonstrated clinically meaningful IOP reductions: mean IOP decreased by −4.2 mmHg at 12–18 months and by −3.4 mmHg at 24–36 months (both *p* < 0.0001). Kaplan–Meier survival analysis confirmed progressive reduction in success rates over time, with 70% at 6 months, 45% at 12 months, and 27% at 24 months. These results suggest attenuation of effectiveness and that re-treatment or augmentation is commonly required. Higher baseline IOP (>21 mmHg) was associated with better success [13].

### 3.3. Safety Profile of SLT

Selective laser trabeculoplasty is generally associated with a favorable safety profile in patients with ocular hypertension and open-angle glaucoma. Across the evidence included in this review, safety signals are dominated by three themes:

(i) An expected post-laser inflammatory response;
(ii) Early IOP spikes;
(iii) Rare but clinically meaningful complications, such as cystoid macular edema (CME).

Consistent with this pattern, both primary studies and the review-level literature support the concept that most SLT-related effects are transient and manageable with appropriate post-procedural monitoring.

Inflammation is a central component of SLT’s safety profile because SLT initiates cellular and molecular changes within the trabecular meshwork. Ayala et al. assessed inflammation after SLT and reported measurable post-treatment inflammatory changes consistent with an acute ocular response [14]. Clinically, this supports the view that inflammation is a known expected consequence of treatment, rather than an unpredictable or progressive complication [14]. Practically, this also implies that inflammation monitoring is particularly relevant in patients who may be more prone to inflammatory responses, since inflammation may influence early post-laser IOP behavior.

Early IOP elevation is another key safety issue because it can drive short-term symptoms and determine the intensity of follow-up needed after treatment. Helvacıoğlu et al. documented early IOP spikes after SLT and analyzed associated risk factors, reinforcing that spikes are not universal but occur frequently enough in a relevant subgroup to justify risk-aware management [15]. Hallaj et al. (2024) further refined early post-SLT IOP behavior by comparing pigmentary glaucoma with primary open-angle glaucoma, suggesting that post-treatment pressure profiles may vary according to underlying clinical context [16]. These findings support structured early follow-up, especially in patients identified as higher risk for IOP spikes.

Although macular complications are not expected as a common outcome, posterior segment events still matter due to their potential visual implications. Örnek et al. described CME following SLT in a patient with ocular hypertension, illustrating that—while rare—clinically significant complications can occur [17]. As a case report, this does not establish incidence, but it confirms that such events remain plausible in post-SLT care. Clinically, it supports prompt assessment of symptoms suggestive of macular edema and the use of imaging when indicated.

Review-level synthesis within the included literature aligns with the interpretation from primary studies. Song reviewed SLT-related complications and emphasized that adverse outcomes are generally limited and often transient, and serious complications are uncommon [18]. Landers similarly reinforced that SLT’s safety profile compares favorably with more tissue-altering approaches, consistent with SLT acting as a selective—rather than destructive—treatment [19].

Overall, SLT’s safety profile can be summarized as follows:

- Acute inflammation occurs after treatment and typically reflects an expected ocular response [14];
- Early IOP spikes may occur in a subset of patients and vary across clinical contexts [15,16,17];
- Rare vision-relevant complications such as CME have been documented in case-level evidence [17];
- Overall complication burden appears low when interpreted through review-level synthesis [18,19].

### 3.4. Treatment Parameters: 360° vs. 180° SLT

A key practical question in SLT is the extent of TM treatment coverage—commonly delivered in either 180° or 360°. Because SLT lowers IOP by initiating biological remodeling within the TM, arc length is relevant not only for the size of early IOP reduction, but also for durability, patient comfort, and feasibility of re-treatment strategies.

In routine care, 360° SLT is typically viewed as providing broader TM coverage, while 180° SLT leaves untreated sectors potentially available for later re-treatment if the initial response is inadequate or weakens over time.

Evidence directly comparing these arcs suggests an advantage for the larger treatment field, particularly during the first year. In a prospective randomized trial, Nagar et al. compared 360° SLT, 180° SLT, and latanoprost in 152 treatment-naïve eyes. At 12 months, 360° SLT produced a mean IOP reduction of approximately 30%, compared with roughly 25% after 180° SLT, while latanoprost achieved a reduction of about 31%. The difference between 360° and 180° was statistically significant (*p* < 0.05), suggesting superior early IOP lowering with full-arc treatment in treatment-naïve eyes [9].

Systematic reviews and meta-analyses support this early advantage. Cunha Amaral et al. synthesized nine studies (1065 eyes) comparing 360° and 180° SLTs and reported consistent benefits favoring 360° at earlier timepoints. In pooled analyses, 360° showed greater IOP reduction at 1 month (MD = −0.99 mmHg, *p* < 0.01), 6 months (MD = −1.37 mmHg, *p* < 0.01), and 12 months (MD = −1.41 mmHg, *p* < 0.01). Success ratios also favored 360° (OR = 1.63, *p* = 0.002). Adverse events were not statistically different between arcs; by 24 months, pooled differences were no longer statistically significant, and heterogeneity seemed driven by one study [20]. Similarly, Zhu et al. meta-analyzed nine comparative studies (1044 eyes) and found a time-dependent pattern, where 360° produced statistically greater IOP reduction at 1 month and 1 year, while at 2 years the difference was no longer statistically significant (*p* ≈ 0.07). Under response criteria (≥20% IOP reduction), 360° also performed better (OR in favor of 360°, *p* = 0.0002 as reported in the extracted summary) [21]. Taken together, pooled results suggest the arc-related advantage is strongest in the short to mid-term, while longer-term differences are less certain.

Although efficacy generally favors 360°, patient-centered tolerability remains an important balancing factor. Larger treatment arcs are consistently associated with potentially greater acute discomfort, even when overall ocular adverse event rates remain broadly similar.

Corneal safety is another practical consideration, especially for patients with limited corneal endothelial reserve. Özen et al. used a prospective bilateral comparative design to evaluate both 180° and 360° arcs in the same patients. They found comparable IOP reduction and no significant difference in central corneal thickness. However, endothelial cell count (ECC) showed a transient reduction at one week after 360° SLT, which resolved by one month—suggesting a short-lived endothelial stress signal without evidence of sustained compromise [22]. Clinically, this supports the general safety of 360° from a corneal perspective in the medium term, while suggesting more caution for patients with constrained endothelial reserve.

Overall, 360° SLT can reasonably be considered a default choice for treatment-naïve OAG and OHT patients when the priority is maximal initial IOP lowering and a higher probability of short-term success. Still, 180° SLT remains useful in selected cases, such as when clinicians aim to preserve untreated sectors for later re-treatment or when tolerability concerns or endothelial reserve issues lead to a more individualized choice. Therefore, the evidence supports a general effectiveness hierarchy (360° > 180° in the first year) moderated by individual patient factors, including comfort expectations and corneal/endothelial status [9,20,21,22].

In summary, randomized and pooled high-level evidence suggests that 360° SLT typically achieves greater IOP reduction and higher success rates than 180° during the first year, while safety is broadly comparable. Over longer follow-up (around 2 years), arc-related differences become less consistent, plausibly reflecting treatment-effect attenuation and heterogeneity across studies. Corneal outcomes appear reassuring, with evidence suggesting endothelial changes after 360° SLT are transient [22].

### 3.5. Repeatability of SLT

A major practical advantage of selective laser trabeculoplasty (SLT) compared with older-generation argon laser trabeculoplasty is the potential for safe re-treatment. This repeatability is grounded in SLT’s tissue-selective mechanism—selective photothermolysis of pigmented TM cells with limited collateral structural damage—which means SLT does not produce the same coagulative scarring that can restrict repeat ALT strategies [23,24,25]. As SLT has increasingly moved earlier in treatment pathways and is frequently used as a first-line option in OAG and OHT, questions about whether subsequent sessions can still lower IOP without compromising safety have become increasingly relevant.

In the re-treatment literature, systematic reviews generally conclude that repeat SLT provides efficacy and tolerability comparable to initial treatment. Guo et al. reviewed repeatability evidence and reported that repeat SLT produces statistically significant IOP reductions, with included series suggesting that the duration of effect after re-treatment may be similar to, or sometimes longer than, the first treatment. Notably, the complication profile after a second SLT session was described as similar to the initial treatment, with adverse effects typically limited to mild discomfort, redness, and transient IOP elevations [26]. Jang et al. performed a systematic review and meta-analysis including 12 studies (nine eligible for meta-analysis). Their quantitative analysis supported a clinically meaningful IOP-lowering effect at 24 months after repeat SLT using a random-effects approach. They concluded that repeat SLT is associated with minimal complications and safety concerns, reinforcing its role as a repeatable therapeutic strategy in POAG [27]. Jang et al. also noted an evidence gap: at the time of their review, data on efficacy after third or later SLT sessions remained insufficient, meaning confidence was strongest for second-treatment outcomes.

Clinically, the strongest signal generally appears in eyes that initially responded to SLT and later experienced loss of effect. Francis et al. presented results from a large multicenter series of 137 patients with primary or secondary OAG who underwent repeat 360° SLT after effect waned. Baseline IOP was similar before SLT1 and SLT2 (20.3 ± 5.2 vs. 19.4 ± 5.0 mmHg). IOP decreased significantly after both procedures, reaching 16.4 ± 3.9 mmHg after SLT1 and 16.7 ± 4.7 mmHg after SLT2 at 12 months. In a matched sub-analysis of 62 eyes with comparable baseline pressures, 1-year IOP was reduced to 16.0 ± 4.3 mmHg after SLT1 and 15.3 ± 3.8 mmHg after SLT2, again demonstrating significant within-baseline reductions without meaningful change in medication burden [24].

Alongside this, the LiGHT trial re-treatment analysis supported favorable efficacy for repeat SLT in medication-naïve eyes. Garg et al. post hoc evaluated repeat SLT in 115 medication-naïve eyes from 90 patients with OAG or OHT. Although absolute IOP reduction at 2 months was slightly greater after initial SLT, adjusting for pre-treatment IOP showed that repeat SLT produced significantly greater IOP reduction. Drop-free IOP control was maintained in 67% of the eyes at 18 months; the duration of the effect was at least equivalent to initial SLT, and no clinically relevant adverse events were reported. Garg et al. (2020) also found no significant difference in repeat-SLT efficacy between eyes needing re-treatment early versus later, consistent with the idea that both early and delayed losses of response may still be addressed with repeat treatment [3].

That said re-treatment should not be restricted to “responders only.” A clinically important question is whether eyes with only modest initial response can benefit from a second SLT session. Khouri et al. examined this in a retrospective study of 51 eyes undergoing repeat 360° SLT. Eyes were stratified by whether the initial response was successful or modest (defined as <20% but >0% IOP reduction). After repeat SLT, treatment success rates did not differ significantly between groups (36.67% vs. 52.38%; *p* = 0.26). Survival analyses similarly showed no meaningful difference in durability, and mean IOP reduction with SLT2 and medication burden changes were also comparable. These results challenge an overly strict assumption that only strong prior responders should be retreated and suggest that a subset of partial responders may still derive meaningful benefit from a second session [28].

More recently, evidence has begun to extend beyond the second re-treatment cycle. Although robust data for many repeat cycles remain limited, Wang et al. reported early outcomes after three sequential 360° SLT treatments in 52 treatment-naïve OAG patients managed with SLT as a stand-alone therapy. IOP reductions were consistent across cycles (approximately 27% after SLT1, 26% after SLT2, and 27% after SLT3), with response rates of 79%, 73%, and 81%, respectively, and no statistically significant differences between cycles. A particularly notable finding was increasing effect duration with successive treatments: mean duration rose from 22.2 months after SLT1 to 33.8 months after SLT2, and remained substantial at 28.9 months after SLT3. Prior response did not predict subsequent response, and no associations were seen with sex, lens status, or glaucoma subtype. These findings suggest that repeat SLT may provide sustained intervals of IOP control across multiple cycles in selected patients [29].

Further, indirect support also comes from longer-horizon first-line SLT cohorts. In a 10-year retrospective study of 108 treatment-naïve eyes with early POAG, Ansari reported high success at 1 year (98%), followed by a decline over time (89% at 5 years and 72% at 10 years). During the 10-year follow-up, 60% of the eyes required re-treatment. The median time to re-treatment was 81 months, and importantly, no eye required incisional glaucoma surgery. This framing supports repeat SLT as an expected and acceptable component of chronic care rather than as a failure endpoint [30]. Consistent with this long-term view, the LiGHT extension allowed a third SLT after year 3 when clinically indicated, with no serious laser-related adverse events reported during long-term follow-up [4].

Taken together, available evidence indicates that repeat SLT preserves the favorable safety profile of the initial procedure. Across the studies by Guo et al. and Jang et al., repeat SLT was mainly associated with mild, transient adversffects similar to primary SLT [26,27]. In the study by Francis et al. (2016), no new safety concerns emerged with SLT [24]. Likewise, Garg et al. reported no clinically relevant adverse events after repeat SLT in medication-naïve eyes, while Wang et al. found no evidence of cumulative toxicity across three treatment cycles [3,29]. This pattern is biologically plausible, as SLT lacks the cumulative thermal destruction and structural scarring that can accumulate with repeated ALT approaches; therefore, repeat SLT remains tissue-sparing across re-treatment cycles [23,24].

From a clinical perspective, overall evidence supports repeat SLT as a safe and effective re-treatment option for OAG and OHT, with strongest support for a second SLT session. Multiple retrospective series and the LiGHT post hoc analyses, together with formal meta-analyses, indicate meaningful IOP reduction with low complication rates [3,24,27]. Emerging data also suggest potential benefit after modest first response, possible clinical usefulness for third sessions in selected patients, and integration into long-term drop-sparing management. Meanwhile, the evidence base is constrained by the predominance of retrospective designs, heterogeneous definitions of “success”, and limited prospective comparative data beyond the second or third cycle. Therefore, repeat SLT should be viewed as a key component of long-term glaucoma care supported by the best availablere-treatment evidence, while recognizing the need for additional prospective studies to clarify optimal timing, patient selection, and durability across repeated treatments.

### 3.6. Predictors of SLT Success

Identifying predictors of SLT success is clinically important for patient selection, counseling, and treatment sequencing. However, the literature remains heterogeneous due to differences in study design, glaucoma subtype, baseline IOP, definitions of success, treatment arc (180° vs. 360°), and follow-up duration. Nevertheless, several themes recur consistently across cohorts—most notably that higher baseline IOP is the most reproducible positive predictor of response.

Across major datasets, baseline IOP stands out as the strongest and most consistent predictor of SLT efficacy. In the study by Kuley et al., which included 997 eyes, eyes meeting predefined success criteria had significantly higher baseline IOP than failure eyes (21.9 ± 5.2 vs. 19.0 ± 5.0 mmHg; *p* < 0.0001) [31]. Hirabayashi et al. likewise identified baseline IOP as the only statistically significant predictor in a cohort of 252 eyes: eyes with baseline IOP > 18 mmHg achieved a mean 6-month reduction of 5.4 ± 5.3 mmHg (23.7%), whereas eyes with baseline IOP ≤18 mmHg paradoxically showed a mean increase of 0.7 ± 4.6 mmHg (*p* < 0.001) [32]. Registry and real-world analyses support this association as well. Elahi et al. in the Lausanne Laser Trabeculoplasty Registry identified higher baseline IOP as an independent positive predictor of success (OR: 1.15 per mmHg; *p* < 0.01) [33], and Khawaja et al. in UK electronic medical record data found baseline IOP > 21 mmHg to be the only clearly significant predictor of success in routine practice [13].

Additional confirmation came from Dahlgren et al. in a post hoc analysis of the Swedish OSLT trial, where baseline IOP predicted both absolute and relative IOP reductions (CE: 0.35; *p* < 0.001 and CE: 0.72; *p* < 0.001, respectively) [34]. This pattern also appears in special populations where absolute pressure lowering is inherently limited. In normal-tension glaucoma (NTG), Lee et al. still found higher pre-SLT IOP to predict success (OR: 3.1; *p* = 0.05) [35]. In a matched study comparing pigmentary glaucoma (PG) versus primary open-angle glaucoma (POAG), Hallaj et al. reported that baseline IOP was the only significant predictor of percentage change in IOP at 12 months (coefficient: 2.154; 95% CI: 0.765–3.543; *p* = 0.003) [36]. Although some of the association may reflect statistical “room to lower,” consistency across independent settings suggests genuine biological relevance.

Beyond baseline IOP, one actionable predictor is the fellow-eye response. This finding supports the hypothesis that individual biological characteristics of the TM influence laser responsiveness. In the Lausanne Registry, Elahi et al. found the strongest predictor of SLT success was the outcome in the contralateral eye (OR: 17.33; *p* < 0.01), substantially outweighing other clinical variables [33]. Bilateral prognostic effects were also described by Shazly and Latina. In bilateral OHT and POAG treated with SLT as the primary therapy, the 3-month percentage of IOP reduction in the first treated eye strongly predicted response in the fellow eye in OHT, with correlations persisting up to 30 months (r = 0.804–0.865; *p* ≤ 0.001), while correlations were weaker and significant only up to 9 months in POAG, yet still suggested prognostic utility [36].

Dahlgren et al. additionally confirmed that the magnitude of IOP reduction in the contralateral eye remained highly significant (CE: 0.57; *p* < 0.001) [34]. Clinically, this implies early treatment response can help inform expectations for the second eye, especially relevant in bilateral OHT and early OAG, where a robust response may support confidence in proceeding, while poor response may prompt earlier consideration of alternative strategies.

#### 3.6.1. TM Pigmentation

TM pigmentation is also a biological variable potentially influencing both efficacy and safety, although its relationship with success is less consistent than baseline IOP. In the Wills Eye retrospective series, Kuley et al. reported that greater angle pigmentation predicted success (*p* = 0.03) [31], and Zhou and Aref summarized evidence supporting the hypothesis that increased pigmentation enhances SLT response, likely due to increased absorption of 532 nm laser energy [37]. By contrast, not all analyses agree. Dahlgren et al. (2026) did not find angle pigmentation to be significantly associated with efficacy [34], and Hirabayashi et al. also reported no significant association between trabecular pigmentation and treatment success [32].

Such discrepancies may reflect differences in pigmentation grading systems, patient ethnicity, delivered treatment parameters, and the dual role of pigmentation in modulating both laser uptake and inflammatory/pressure responses. This dual interpretation becomes particularly relevant in pigment-dense glaucoma. Hallaj et al. showed that IOP spikes occurred exclusively in PG eyes (7.6% vs. 0% in matched POAG controls), despite PG eyes receiving significantly lower total laser energy [16]. This supports a melanin-load mechanism rather than a pure dose effect. Helvacıoğlu et al. similarly demonstrated that heavy angle pigmentation and darker iris color were strongly associated with greater early IOP spikes (both *p* = 0.0001), although they were not necessarily predictors of better long-term efficacy [15]. Taken together, pigmentation may therefore be bidirectional: moderate pigmentation may favor response by improving laser absorption, whereas very heavy pigmentation may increase the probability of adverse inflammatory or pressure responses that could overshadow long-term benefits.

#### 3.6.2. Glaucoma Subtype

Glaucoma subtype can shape the balance between expected benefit and durability. In NTG, absolute IOP lowering is limited, but SLT can still produce clinically relevant percentage reductions. Lee et al. reported a success rate of 61.7% and a mean 1-month IOP reduction of 21.5 ± 11.4% [35]. In pseudoexfoliative glaucoma (PXFG), newer multivariable data are less favorable. Dahlgren et al. found PXFG to be a negative predictor of efficacy (CE: −0.69; *p* = 0.02), suggesting early reductions may occur but durability may be inferior [34]. In PG, overall efficacy may be comparable to POAG, but safety signals differ. Hallaj et al. reported no significant difference in 12-month failure rate compared with POAG (50.7% vs. 43.1%; *p* = 0.205) yet spike risk was clearly heightened [16]. Accordingly, subtype may influence how efficacy and tolerability trade-offs present clinically, rather than success alone.

#### 3.6.3. Age

Age-related prediction appears inconsistent across studies. Ayala and Chen reported that increasing age was negatively correlated with time to failure (*p* = 0.015), implying reduced durability in older patients [14]. The authors speculated that this might relate to age-related changes in biological remodeling responses. In contrast, Kuley et al., Hirabayashi et al., and Dahlgren et al. did not find independent effects of age [31,32,34]. Lee et al. observed an age–success association only in univariate analysis that did not persist in multivariate modeling [35]. Overall, age alone does not appear sufficiently reliable for selection, though it may interact with disease chronicity, subtype, and biological response heterogeneity.

#### 3.6.4. Medication Status

Medication status and treatment burden also emerge as clinically relevant—though biologically complex—predictors. Elahi et al. in the Lausanne Registry found prior medical treatment before SLT positively associated with success (OR: 2.57; *p* = 0.03), which may partly reflect how success was defined (including medication reduction in “qualified success”) [33]. In contrast, Dendumrongsup, using mechanistic modeling, proposed that medications that increase uveoscleral outflow—especially prostaglandin analogs—might reduce the relative SLT effect by decreasing reliance on trabecular outflow [38]. This theoretical framework is consistent with clinical observations where some studies show stronger SLT performance in medication-naïve eyes, including paradigms like LiGHT.

Further evidence from special populations supports the importance of medication burden as a marker of response complexity. In NTG, Lee et al. found that use of three or more glaucoma medications predicted treatment failure on univariate analysis (OR: 0.1; *p* = 0.02) [35]. In POAG from the same group, multiple medications and topical carbonic anhydrase inhibitor use were associated with success on univariate analysis but did not remain independent predictors in multivariate models [35]. Overall, pre-existing medication burden may serve to identify patients with more challenging disease, but the direct biological interaction between medical therapy and SLT efficacy remains incompletely resolved.

### 3.7. Real-World Outcomes and Special Populations

Real-world effectiveness of SLT matters because routine care encompasses a wider spectrum of disease severity, variable adherence and tolerability of topical therapy, different re-treatment behaviors, and success definitions that are often less standardized than in RCTs. In observational studies, SLT typically results in clinically meaningful IOP reduction, but durability often declines sooner in less controlled settings.

In the UK’s electronic medical record (EMR)-based practice, Khawaja et al. analyzed 831 eyes treated with SLT at five tertiary centers and showed significant IOP lowering after initial treatment. Mean IOP decreased by 4.2 mmHg from a baseline of 22.0 mmHg at 12–18 months and by 3.4 mmHg at 24–36 months (both *p* < 0.0001) [13]. Medication use increased modestly over time, consistent with pharmacological reintensification as SLT effects waned. Specifically, there were small but significant increases in the number of drops at 12–18 months (+0.13; *p* = 0.007) and at 24–36 months (+0.20; *p* = 0.005). Kaplan–Meier estimates showed progressive loss of “success” over time, with estimated success rates of 70% at 6 months, 45% at 12 months, and 27% at 24 months. Taken together, these findings support the pragmatic view that SLT is effective but that re-treatment and escalation may emerge earlier in real-world populations than in trial cohorts.

Consistent with this, Khawaja et al. support baseline IOP as an important predictor of outcomes. In his dataset, higher baseline IOP (>21 mmHg) dominated as predictor of success (HR: ~0.67; *p* < 0.001), while age, visual field severity, and concurrent medication use did not predict success [13].

Alongside predictors, real-world treatment sequencing has also been explored to help clinicians understand where SLT fits across therapeutic pathways. Fu et al. followed 2708 patients (4564 eyes) treated across a 10-year period in the UK’s secondary and tertiary services. They compared multiple pathways, including initiation with drops, initiation with SLT, escalation to additional drops, and escalation from one drug to SLT plus one drug [39]. Using Kaplan–Meier methods and a failure definition that included insufficient IOP reduction, IOP thresholds, increased medication burden, re-treatment need, or incisional glaucoma surgery, the median time to treatment failure differed across strategies: 1.72 years for single topical agent initiation, 1.35 years for SLT initiation, 1.32 years for escalation to two topical agents, and 1.53 years for escalation from one drug to SLT plus one drug [39]. The authors also acknowledged the limitation of non-randomized design: comparative effectiveness (SLT vs. drops) cannot be interpreted causally due to confounding by indication. Nonetheless, observationally relevant implications remain: adding SLT to existing medication may prolong time to failure compared with intensifying medication alone, within the constraints of real-world evidence [39].

Moreover, these real-world considerations extend beyond patient and disease factors to delivery models. As healthcare systems adapt to growing demand and SLT becomes recommended more often as a first-line option, optometrist-delivered SLT has become a feasible strategy when integrated into structured clinical governance. Lee et al. compared optometrist- versus ophthalmologist-delivered SLT across three hospital eye units in a multicenter retrospective observational study, following 207 eyes (131 patients) for up to 24 months [40]. No statistically significant differences were identified between delivery groups for IOP reduction, visual acuity change, or medication burden at any timepoint (all *p* > 0.05) [40]. Differences instead appeared in downstream management: more optometrist-treated eyes proceeded to glaucoma surgery, while ophthalmologist-treated eyes had higher drop burden and a higher composite failure risk up to 18 months [40]. Lee et al. interpreted these patterns as reflecting differences in escalation strategies rather than differences in SLT effect [40].

To support this approach, Konstantakopoulou et al. described a structured training protocol for optometrist-delivered SLT and reported early service evaluation findings consistent with safe delivery when training and local protocols are followed [41]. In addition, Swystun et al. provides valuable large-scale estimates of safety and effectiveness; retrospective designs limit causal inference about which elements of practice—such as variability in laser technique, medication adjustment patterns, or follow-up timing—influence outcomes most [42]. Overall, these data support multidisciplinary and community-integrated delivery pathways, consistent with guideline momentum (e.g., NICE NG81) while addressing service bottlenecks in high-demand healthcare environments [43].

Within special populations, however, SLT can remain appropriate but may require tailored risk stratification. In exfoliative glaucoma—including pseudoexfoliative glaucoma (PXFG)—SLT has been described as effective overall, but heavier TM pigmentation may plausibly increase the risk of early IOP spikes. Clinically, this does not suggest avoiding SLT, but rather applying careful prophylaxis and closer post-procedure monitoring when pigmentation burden is substantial, consistent with broader evidence linking pigmentation to acute pressure responses.

In pigmentary glaucoma (PG), Hallaj et al. presented a key matched safety analysis comparing PG outcomes with matched POAG controls while noting that PG eyes received lower total laser energy. Despite clinician-aware dose adjustment, IOP spikes occurred exclusively in PG eyes (5/65; 7.6%) and in none of the POAG eyes (0/65; *p* = 0.058) [16]. Long-term efficacy was broadly comparable, with 12-month failure rates of 50.7% in PG versus 43.1% in POAG (*p* = 0.205). Multivariable modeling identified pre-laser IOP as the key predictor of percentage change in IOP rather than glaucoma type [16]. Thus, in pigmentary diseases the main clinical emphasis shifts toward recognizing and mitigating spike risk rather than assuming inherently inferior long-term efficacy.

In normal-tension glaucoma (NTG), absolute IOP lowering is limited by baseline pressures near normal ranges. Even so, SLT can still achieve clinically meaningful percentage reductions and reported success rates above 60% in selected cohorts, most importantly, particularly when higher pre-SLT IOP and early IOP response predict durability. Finally, eyes with previously filtered or surgically altered outflow pathways may represent a more complex target for trabeculoplasty. Outflow dynamics and TM function may be altered, and clinical series often suggest reduced success rates. In such cases, SLT may be best positioned within a tailored strategy rather than as a universal default in all post-surgical scenarios.

Despite its overall safety, rare posterior segment inflammatory events—including cystoid macular edema (CME)—have been described, particularly in patients with prior intraocular surgery. For example, Örnek et al. reported CME occurring one week after 360° SLT in a pseudophakic patient with traumatic cataract surgery history; the episode resolved fully following topical corticosteroid and NSAID therapy and did not recur on follow-up [17]. Although such events remain rare, they strengthen the rationale for documenting surgical history and maintaining a low threshold for OCT assessment if vision decreases after SLT [17].

In summary, routine effectiveness generally follows the direction of trial efficacy but durability often declines faster in practice, with lower success rates at 24 months than in RCT estimates. Higher baseline IOP repeatedly predicts better SLT success. Trained optometrist-delivered SLT can yield comparable outcomes when embedded within structured service models. In pigmentary glaucoma, long-term efficacy appears similar to POAG, but acute IOP spike risk is clearly higher. Rare complications such as CME cluster in higher-risk contexts, particularly those involving prior intraocular surgery [13,16,17,40].

### 3.8. Emerging Laser Modalities: DSLT and MLT

Beyond conventional SLT, recent years have brought increased interest in alternative laser trabeculoplasty approaches designed to improve workflow efficiency, patient comfort, and acute procedural tolerability. Alongside established SLT, two emerging modalities—direct selective laser trabeculoplasty (DSLT) and micropulse laser trabeculoplasty (MLT)—are positioned as strategies that may improve the balance between effectiveness and procedure-related burden. Although SLT remains the best-established option, growing evidence for DSLT and MLT increasingly informs how laser therapy could be delivered and tolerated in routine glaucoma care.

DSLT is a technological evolution intended to reduce operator dependence and expand access by simplifying the delivery process. Unlike conventional SLT, which requires gonioscopic visualization and manual spot placement, DSLT is delivered using a non-contact automated translimbal approach guided by eye tracking. This removes the need for slit-lamp gonioscopy and standardizes treatment delivery, lowering the expertise barrier associated with conventional laser trabeculoplasty. Early prospective evidence from Goldenfeld et al. supported the feasibility and safety of automated DSLT, demonstrating clinically relevant IOP reduction without serious adverse events and providing dose–response information to support energy selection [44].

Among the strongest comparative framework to date is the GLAUrious trial. Congdon et al. described a multicenter randomized investigator-masked non-inferiority evaluation of DSLT versus conventional SLT, using a prespecified margin for mean IOP change at 6 months [45]. Although the 6-month primary endpoint was not formally non-inferior in the published summary, subsequent exploratory comparisons at 12 months supported DSLT as an effective option with similar efficacy between groups [45]. Safety signals were consistent with a mostly mild ocular adverse event profile, and punctate subconjunctival hemorrhage was described as more frequent in the DSLT group without major clinical consequences [45].

In addition to controlled trial evidence, retrospective real-world data have begun to emerge. Zollet et al. reported broadly similar 12-month survival outcomes between DSLT and conventional SLT in a UK tertiary-center comparison, with no statistically significant between-group differences, though limited by small sample size and the retrospective design [46]. Overall, available findings support the plausibility that automated DSLT can achieve comparable clinical outcomes while improving delivery standardization in settings where workflow constraints limit conventional SLT implementation.

MLT is designed to reduce thermal tissue injury while preserving biologically active stimulation for trabecular outflow. By delivering energy in micropulsed intervals interspersed with cooling periods, MLT aims to mitigate collateral heating effects that may contribute to acute tolerability concerns seen with other laser approaches. Mechanistically, MLT has been described as promoting trabecular meshwork stimulation through cytokine-related pathways and extracellular matrix remodeling, with the expectation of fewer IOP spikes and less anterior segment inflammation compared with conventional SLT.

Clinical viability is supported by synthesis-level evidence. Confalonieri et al. conducted a PRISMA-guided systematic review of 15 studies in POAG, reporting clinically meaningful IOP reductions alongside a generally favorable safety profile characterized mainly by mild and transient adverse events [47]. Moreover, comparative meta-analytic data also suggest that SLT may produce modestly greater early IOP reduction at 1 and 6 months, with differences converging by 12 months, while MLT shows a statistically significant reduction in post-laser IOP spikes [47]. Similar conclusions were reported by Al-Wizni et al, who found MLT non-inferior to SLT for efficacy outcomes within the first year, with no significant differences in pooled success or IOP reduction metrics, while noting that the overall dataset remains smaller than for SLT [48].

Head-to-head cohort evidence adds further nuance. In a retrospective comparative study, Pimentel et al. reported comparable 12-month IOP reduction and success rates between SLT and MLT, with differences emerging particularly in medication burden—suggesting that secondary outcomes may depend on baseline treatment strategies and local clinical practices [49]. Overall, these results support a pragmatic approach where selection between MLT and SLT can reasonably consider acute spike risk, patient tolerability priorities, and the trade-off between the magnitude of early IOP lowering and spike-oriented safety outcomes.

From a clinical positioning standpoint, DSLT mainly targets standardization and access through automated, non-contact translimbal delivery, whereas MLT mainly targets tolerability through micropulse delivery and cooling intervals. Current comparative findings indicate that both modalities can deliver clinically relevant IOP lowering. DSLT is supported by comparative trial infrastructure and growing corroboration; MLT is supported by systematic review and meta-analytic evidence suggesting comparable 1-year efficacy to SLT alongside a potentially more favorable post-laser spike profile. Nonetheless, both DSLT and MLT currently have important limitations in terms of long-term clinical maturity. In contrast to conventional SLT—supported by a substantially larger and longer-running evidence base—available randomized and comparative data for DSLT and MLT predominantly inform outcomes over the first 12–24 months, with durability, re-treatment frequency, and long-term treatment sequencing remaining less well characterized. In particular, it is still unclear how early differences in tolerability (e.g., reduced IOP spikes for MLT) translate into sustained disease control over multiple re-treatment cycles, as well as how these modalities may influence medication escalation patterns over time. In conclusion, the current evidence supports DSLT and MLT as clinically promising alternatives, but continued follow-up in longer-term randomized or well-designed prospective studies is needed before they can be considered fully equivalent to conventional SLT across the entire glaucoma treatment horizon.

### 3.9. Cost-Effectiveness of SLT

From a health-policy standpoint, the key economic question is whether selective laser trabeculoplasty (SLT) can provide comparable (or superior) IOP control to topical therapy while lowering overall costs, including downstream expenses related to clinic visits, medication use, and—depending on the healthcare system—later surgical escalation. Notably, evidence for direct cost-effectiveness is strongest when SLT is evaluated as first-line therapy, because this is where the balance between an upfront laser cost and longer-term medication avoidance is most favorable.

The key randomized controlled trial addressing this question in routine clinical settings was the multicenter LiGHT trial. LiGHT compared SLT with eye drops for treatment-naïve patients with open-angle glaucoma (OAG) or ocular hypertension (OHT). In a pragmatic observer-masked design, patients were randomized to initial SLT or initial topical therapy, with follow-up at 36 months and economic evaluation from an ophthalmology perspective. The trial however, found no meaningful difference in generic health-related quality of life (mean EQ-5D at 3 years: 0.89 vs. 0.90). In terms of clinical outcomes, findings favored SLT in terms of “drop freedom”: a total of 74.2% of patients in the SLT arm required no eye drops to maintain IOP at target over 36 months, while no patients in the SLT group required surgery to lower IOP versus 11 patients in the eye-drop group [7].

From an economic evaluation perspective, LiGHT reported that from an ophthalmology cost perspective SLT had a 97% probability of being more cost-effective than eye drops first at a willingness-to-pay threshold of £20,000 per QALY gained [7]. These findings support SLT as a cost-effective early disease-pathway strategy and align economic value with patient-centered outcomes such as reduced medication burden and improved IOP stability.

In addition to the UK NHS context, additional economic modeling and payer-perspective analyses support the same general conclusion: SLT often dominates (less costly and at least as effective) or becomes cost-effective over relatively short horizons, especially when SLT is used upfront and repeated when clinically needed. To illustrate this, in a Canadian payer perspective Markov model evaluating newly diagnosed OAG over a 20-year horizon, SLT was associated with lower healthcare system costs and higher QALYs compared with topical medication, leading to dominance in the base case. Sensitivity analyses supported robustness across discount rates and key parameter variations [50]. However, although long-term uncertainty remains—particularly around utilities and real-world adherence—these modeling results still support that SLT may be economically advantageous over patients’ lifetimes rather than only within short trial durations.

Across other health systems with different reimbursement structures, SLT has also been evaluated as a cost-saving alternative to continuous medication. In Brazil’s Unified Health System (SUS), Barbosa et al. assessed cost-effectiveness using real-world clinical data and found SLT to be a dominant strategy compared with continuous hypotensive drops in mild-to-moderate OAG over a one-year horizon. The intervention group showed similar (and slightly greater) IOP reduction compared with the reference group while incurring substantially lower annual medication costs; the reported ICER was negative (−R$1.82 per unit of effectiveness, defined as mmHg reduction), indicating more benefit at lower cost [51]. While a one-year horizon limits interpretation regarding long-term durability and re-treatment frequency, the direction of effect supports the broader economic logic of SLT—shifting from recurring medication costs to a one-time (or periodically repeatable) laser procedure—translating across payer systems.

Likewise, additional evidence also suggests that affordability and “time-to-cost parity” can be relevant for broader access discussions. A US cost analysis estimated the “time threshold” at which bilateral SLT becomes less costly than bilateral topical therapy. Under conservative assumptions—including probabilistic acute adverse event management—SLT was estimated to become less costly than generic latanoprost after 13.1 months [52]. However, such estimates depend on drug pricing and adverse event management costs; the practical implication is that even from a cost-minimization perspective SLT can reach cost equivalence with medications on a relatively short timescale, particularly when generic prices remain low and adherence barriers are clinically relevant [52].

At the same time, macro-level affordability comparisons make the economic case even stronger in settings where medication access and long-term affordability are major constraints. A worldwide pricing comparison found substantial cross-country variability in affordability of glaucoma medications, laser trabeculoplasty, and trabeculectomy relative to median household income. In many countries, annual therapy with prostaglandin analogs or multiple medication classes impose a financial burden exceeding affordability thresholds, and laser trabeculoplasty costs less than prolonged medication in a substantial proportion of studied countries [53]. Importantly, affordability studies do not equal cost-effectiveness (because they do not measure QALYs or adherence-adjusted outcomes); they provide important contextual justification: SLT may reduce economic burden in settings where recurring medication costs are prohibitive.

Taken together, across randomized evidence, long-horizon modeling, real-world cost-effectiveness evaluations, and cost-parity analyses [7,50,51,52], the overall conclusion is consistent: SLT is cost-effective, and often cost-saving, when used as first-line therapy. The main driver is reduced (or delayed) ongoing topical medication use, while maintaining clinical effectiveness and improving the likelihood of sustained target IOP control.

## 4. Discussion

In synthesis, the results indicate that SLT functions as a tissue-sparing, biologically mediated intervention with a mechanistic plausibility supported by emerging laboratory evidence. Clinically, its efficacy is consistently demonstrated in the short-to-medium-term study, aligning well with randomized controlled trial expectations. However, durability in real-world practice appears less robust than what is often reported in tightly controlled trial settings—suggesting that effectiveness may decline earlier in routine care, particularly when follow-up, re-treatment timing, and treatment escalation are not as standardized. Accordingly, SLT should be viewed not as a “one-off cure,” but as a management strategy whose real impact depends on how it is integrated into the overall glaucoma treatment pathway.

From a safety standpoint, the evidence is generally reassuring. The most commonly observed issues include transient post-laser inflammation and early IOP rises (including occasional symptomatic episodes), while rare posterior segment complications such as macular edema have been reported infrequently. Notably, the findings support the notion that SLT is not “risk-free”—rather, it is a procedure where safety is optimized through structured monitoring and appropriate peri-procedural management, especially in patients at higher risk for pressure spikes.

With respect to treatment delivery, the extent of ablation shows a clinically relevant early effect. In general, 360° SLT tends to produce a greater early IOP reduction compared with 180°, with differences narrowing over time. This, in turn, supports the practical interpretation that treatment selection should not be driven solely by the goal of maximal immediate IOP lowering, but also by tolerability, patient-specific risk, and the practical realities of re-treatment planning (including the possibility of treating previously untreated areas).

A central strength of SLT highlighted across the evidence is re-treatment/replication of effect. The mechanistic rationale and clinical outcome patterns support repeatability, with re-treatment typically demonstrating a comparable safety profile and clinically meaningful IOP lowering for many patients. At the same time, evidence becomes less certain for third (and later) treatment sessions, where data are still more limited and heterogeneous, underscoring the need for further research to clarify optimal timing and patient selection beyond the second course.

Similarly, predictors of success are also broadly consistent: higher baseline IOP is repeatedly associated with better outcomes. Other factors—including pigmentation, age, and glaucoma subtype—introduce variability in both efficacy and tolerability, particularly influencing the likelihood of early IOP spikes and the stability of response. Specifically, pigmentary glaucoma and normal-tension glaucoma show more variable benefit, reinforcing the importance of careful selection and closer post-procedural surveillance rather than assuming uniform response across subtypes.

Promising findings across different eye-care professional groups suggest that SLT can be implemented beyond ophthalmology and can produce clinically meaningful IOP lowering. Nevertheless, service-delivery evidence should be interpreted cautiously. Much of the optometrist-versus-ophthalmologist comparison relies on retrospective observational designs, which are vulnerable to non-random differences in patient selection and escalation strategies. Consequently, this creates a risk of confounding by indication: clinicians may choose SLT for different patient profiles or at different points in the pathway based on perceived risk, comorbidities, stability versus progression, expected response, medication tolerance, and practical follow-up considerations, even when statistical adjustment for measured baseline factors (e.g., initial IOP or treatment burden) and unmeasured influences—such as adherence to topical therapy, clinical judgment, or socioeconomic effects on appointment attendance—can bias comparisons.

Another limitation is how “effectiveness” is defined in real-world studies. Many use composite endpoints that combine IOP response with pathway-dependent events (medication changes, re-treatment, additional procedures, and referral for surgery). Such endpoints are clinically relevant for system planning, but they are also strongly influenced by post-procedural clinical decision-making (reassessment timing, interpretation of IOP trends, and escalation thresholds). As a result, differences in observed outcomes may reflect monitoring and escalation behavior as much as (or more than) differences in the laser’s intrinsic effect.

Despite the overall direction of benefit aligning with RCT evidence, some service-delivery studies suggest shorter apparent durability. This does not necessarily contradict trial efficacy; rather, real-world outcomes may vary with follow-up timing, re-treatment triggers, and differences in how quickly thresholds are applied.

Finally, feasibility/protocol adherence studies support early deliverability, but they often do not fully resolve the policy-critical questions: long-term durability, stability of practice over time, and generalizability across settings with different patient mix, resources, and governance maturity. Implementation early in a service’s learning curve may look different from later phases when training and audits are embedded.

The most defensible interpretation is not that provider types are interchangeable in laser efficacy, but that SLT can be delivered safely and meaningfully when embedded within an integrated, standardized, and audited glaucoma management pathway. Scaling requires explicit governance of patient selection, monitoring cadence, escalation criteria, re-treatment rules, and referral processes—not only procedural training. Where outcomes are assessed using pathway-sensitive composite endpoints, causal equivalence across providers should not be assumed.

Retrospective observational design, confounding by indication, composite endpoint sensitivity to pathway behavior, variable documentation and follow-up completeness, and uncertainty about long-term generalizability and durability—particularly beyond the early implementation period—should be considered.

Overall, service-delivery findings provide reassuring real-world signals consistent with RCTs for meaningful short-to-medium-term benefit, but they must be interpreted as evidence of workability within systems, not definitive proof of equivalent long-term laser effectiveness across provider groups. Continued monitoring, replication, and stronger prospective or quasi-experimental designs with clearer separation of IOP efficacy from re-treatment/escalation events are needed.

In addition, emerging laser modalities—particularly DSLT and MLT—offer promising early results, especially in tolerability and workflow feasibility. Nonetheless, current evidence remains limited by shorter follow-up windows, typically within 12–24 months, which means durability and re-treatment patterns are not yet well established. For these newer approaches, longer-term outcome data are essential before firm conclusions about sustained disease modification can be drawn.

From a policy and economic standpoint, the evidence supports the expansion of SLT, particularly when used earlier in the treatment pathway. By reducing reliance on chronic medication and potentially delaying escalation, SLT appears economically favorable in multiple models—though the magnitude of benefit depends on local pathways, uptake, and monitoring practices.

SLT emerges from this evidence base as an effective, generally safe, and repeatable intervention within a broader glaucoma management strategy. Its real-world performance, however, depends critically on structured implementation—training, governance, standardized follow-up, and audit—and on careful patient selection tailored to risk (including the likelihood of early IOP spikes and variable response in certain subtypes). Long-term durability remains an under-characterized area, and ongoing research—especially for repeat treatments beyond the second session and for newer modalities like DSLT/MLT—remains necessary.

## 5. Conclusions

This non-systematic narrative review synthesizes evidence up to 31 July 2026 on selective laser trabeculoplasty (SLT) for open-angle glaucoma (OAG) and ocular hypertension (OHT). Overall, the reviewed data support SLT as an effective and generally safe procedure-based approach to lower intraocular pressure (IOP), with benefits extending beyond short-term IOP reduction into meaningful long-term disease control in appropriately selected patients.

Mechanistic insights indicate that SLT acts mainly through selective photothermolysis of pigmented trabecular meshwork cells, followed by downstream biological and remodeling processes that enhance aqueous outflow while limiting collateral structural damage. This tissue-sparing profile provides a strong rationale for repeatability. Across repeat-treatment studies and systematic syntheses (and in selected contexts further), a second SLT session can deliver clinically relevant IOP lowering with adverse events that are predominantly mild and transient.

The strongest clinical efficacy evidence comes from the LiGHT program, where SLT-first therapy demonstrated comparable or superior IOP control compared with initial topical eye drops, improved “drop-free” persistence, reduced medication burden, and reduced longer-term need for incisional surgery. However, durability declines over time in both trial and real-world settings, reinforcing the need for individualized follow-up and escalation planning.

Predictors of response are consistently dominated by baseline IOP, while additional factors—such as early fellow-eye response, glaucoma subtype, and angle pigmentation—may influence both the magnitude of effect and the risk of early IOP spikes.

Safety findings show that most post-SLT events (particularly anterior chamber inflammation and early IOP elevations) are expected and manageable. Rare but vision-relevant complications, such as cystoid macular edema, have been reported mainly in higher-risk contexts (e.g., eyes with prior intraocular surgery). Comparative evidence suggests that 360° SLT generally provides stronger early IOP lowering than 180° SLT. Beyond early periods, arc-related differences become less consistent, and patient-centered tolerability remains important.

Economic and service-delivery evidence converge to support SLT as a cost-effective first-line strategy, especially when it reduces ongoing topical medication use. Emerging modalities—automated direct SLT (DSLT) and micropulse laser trabeculoplasty (MLT)—are promising for workflow efficiency and tolerability optimization. Nevertheless, the current evidence base is smaller than for conventional SLT, with key uncertainties remaining regarding long-term durability and re-treatment patterns. Collectively, these findings position SLT as a cornerstone of modern glaucoma care, provided implementation incorporates patient selection, parameter choice, risk-adapted follow-up, and safe re-treatment strategies while emerging technologies continue to be evaluated in real-world settings.

## Figures and Tables

**Table 1:** Evidence distribution by study type and time period.

Study Type	Jan 2019–Jul 2026	≤2018 *	Total
RCT/prospective randomized study/investigator-masked RCT	7	0	7
Systematic review (incl. update/narrative systematic) + meta-analysis/network meta-analysis	11	1	12
Review (narrative/overview/literature review)	5	1	6
Guidance/professional organization report (incl. NICE)	2	0	2
Observational/real-world analysis	9	0	9
Observational study (cohort/clinical comparative/assessment, etc.)	4	0	4
Retrospective observational study/retrospective comparative	2	0	2
In vitro experimental/laboratory study	0	1	1
Methodological/modeling/feasibility/repeatability	1	1	2
Economic evaluation: cost-effectiveness/health economics/cost comparisons	3	2	5
Prospective clinical study (pilot/first-in-human concept)	1	0	1
Total	45	8	53

* Studies published in or before 2018.

## Data Availability

No new data were generated or analyzed in this review. Data sharing is not applicable to this article.

## Abbreviations

ALT	Argon laser trabeculoplasty
CME	Cystoid macular edema
DSLT	Direct selective laser trabeculoplasty
ECC	Endothelial cell count
EQ-5D	EuroQol 5-Dimension
IOP	Intraocular pressure
LiGHT	The Laser in Glaucoma and Ocular Hypertension
MLT	Micropulse laser trabeculoplasty
NTG	Normal-tension glaucoma
OAG	Open-angle glaucoma
OHT	Ocular hypertension
OCT	Optical coherence tomography
PGA	Prostaglandin analog
PG	Pigmentary glaucoma
POAG	Primary open-angle glaucoma
QALY	Quality-adjusted life year
RCT	Randomized controlled trial
PXG	Pseudoexfoliative glaucoma
SC	Schlemm’s canal
TM	Trabecular meshwork
